# A Novel MMP-2 Inhibitor 3-azidowithaferin A (3-azidoWA) Abrogates Cancer Cell Invasion and Angiogenesis by Modulating Extracellular Par-4

**DOI:** 10.1371/journal.pone.0044039

**Published:** 2012-09-04

**Authors:** Bilal Rah, Hina Amin, Khalid Yousuf, Sheema Khan, Gayatri Jamwal, Debaraj Mukherjee, Anindya Goswami

**Affiliations:** 1 Molecular Signal Transduction Laboratory, Cancer Pharmacology Division, Indian Institute of Integrative Medicine (CSIR), Jammu Tawi, India; 2 Nautral Product Chemistry, Indian Institute of Integrative Medicine (CSIR), Jammu Tawi, India; Southern Illinois University School of Medicine, United States of America

## Abstract

**Background:**

Withaferin A, which is a naturally derived steroidal lactone, has been found to prevent angiogenesis and metastasis in diverse tumor models. It has also been recognized by different groups for prominent anti-carcinogenic roles. However, in spite of these studies on withanolides, their detailed anti-metastatic mechanism of action remained unknown. The current study has poised to address the machinery involved in invasion regulation by stable derivative of Withaferin A, 3-azido Withaferin A (3-azidoWA) in human cervical HeLa and prostate PC-3 cells.

**Methods and Principal Findings:**

Sub-toxic concentration of 3-azidowithaferin A (3-azido WA) inhibited cancer cell motility and invasion in wound healing and Boyden chamber invasion by suppressing MMP-2 activity in gelatin zymography and its expression has proved to be a major obstacle in chemo-sensitivity. We have uncovered a novel mechanism of 3-azidoWA induced extracellular pro-apoptotic candidate tumor suppressor Par-4 protein stimulation in conditioned media and also noticed a concomitant marked reduction in pAkt and pERK signaling by immunoblot analysis. Furthermore, our zymography results suggest 3-azidoWA induced MMP-2 inhibition was mediated through secretory Par-4. The inhibition of apoptosis by 3-azidoWA could not restore MMP-2 gelatinase activity. In addition to this, our *in vivo* animal experiments data showed 3-azidoWA abrogated neovascularisation in dose dependent manner in mouse Matrigel plug assay.

**Conclusion/Significance:**

For this report, we found that 3-azidoWA suppressed motility and invasion of HeLa and PC-3 cells in MMP-2 dependent manner. Our *in vitro* result strongly suggests that sub-toxic doses of 3-azidoWA enhanced the secretion of extracellular Par-4 that abolished secretory MMP-2 expression and activity. Depletion of secretory Par-4 restored MMP-2 expression and invasion capability of HeLa and PC-3 cells. Further, our findings implied that 3-azidoWA attenuated internal phospho-ERK and phospho-Akt expression in a dose dependent manner might play a key role in inhibition of mouse angiogenesis by 3-azidoWA.

## Introduction

Extracellular secretory pathways are considered to play pivotal role in human physiology. Body’s vital hormones and growth factors are secreted and they control the development and differentiation of organs in normal physiological condition. Likewise, systemic (extracellular) proteins attribute major *in vivo* function during tissue growth and apoptosis [1]. Prostate apoptotic response 4 (Par-4) is ubiquitously expressed and evolutionary conserved pro-apoptotic protein whose expression was mainly correlated with the cells that undergo apoptosis due to exogenous insults [2]. Apart from its intracellular function, the new perspective of extracellular secretion in different cancer cells has augmented the therapeutic potential of Par-4 [3]. Recently, Burikhanov et al. have shown that mammalian cells in general caused secretion of Par-4. However, the apoptotic induction by extracellular Par-4 occurring via cell- surface GRP-78 was found to promote cell invasion and tumorigenesis [3]. The stabilization of pro-angiogenic GRP-78 by Par-4 has been designated an anti-invasive role of extracellular Par-4.

Metastasis is a multi-step process involving cell migration and pericellular proteolysis of ECM that mediates cancer cells protrusion [4]. Matrix metalloproteinases (MMP’s) are responsible for the degradation of environmental barriers, such as the extracellular matrix and basement membrane [5], [6]. Amongst the MMP family members, MMP-2 and -9 are generally considered to be the malignancy of various tumors as well as poor prognosis of many cancers [6]. Thus, MMP’s are capable of cleaving type IV basement membrane collagen (MMP-2 and -9) and add value for drug development. Compelling preclinical studies from diverse laboratories have provided overwhelming support for direct relationship between MMP-2 over expression and tumor invasion/metastasis [7], [8]. During the developmental phase, many of the MMP inhibitors failed in the early phase clinical trials because of extensive homology between catalytic domains of MMP’s. Moreover, most of the synthetic/semi-synthetic inhibitors of MMP’s were withdrawn during clinical trials due to unanticipated long term drug intolerance reduced drug compliance [9]. On the other hand, recently, natural products or natural product derivatives have been considered extremely potential to abrogate MMP-2 and -9 mediated invasion/metastasis either *in vitro* or *in vivo* set up. These include aqueous cinnamon extract [10], green tea extract [11], curcumin [12], and steroidal saponin from fenugreek [4], chitooligosacharides (COS) from marine natural products [13].

Withaferin A (WFA) is a prototype of the withanolide class of natural products that exhibit diverse pharmacological activities, including antitumor, antiangiogenic, cardioprotective, anti-inflammatory, and immunomodulatory effects [14], [15]. The bioactive properties of Withaferin A includes cytoskeletal remodeling by binding to Annexin II [16], antiangiogenic [17], [18] and antitumor activity [19], [20] by inhibition of proteasomal chymotrypsin [21] and apoptotic induction by inhibition of protein kinase C [22]. Recently, Oh et al have demonstrated the caspase-3 activation through Withaferin A [23]. Apart from its anti-cancerous activity, Withaferin A has also been documented for its anti-inflammatory property by suppressing alpha-2-macroglobulin [24]. With our recent success towards the development of a library of Withaferin A semisynthetic analogues, the rational screening strategy lead to the generation of 3-azidoWA, the potent anticancer candidate [25]. Although the importance of α-β-unsaturated functionality of ring A of Withaferin A and the anticancer potential of 3-azidoWA became obvious, still its mode of action was not clear.

In this study we evaluated the mechanistic role of 3-azidoWA (3-azido WA), an azido Withaferin derivative on motility and invasion of cancer cells. We also wanted to co-relate this study with the signaling pathways *viz*, ERK, Akt, which are activated in different cancers and potentiates invasion and metastasis of diverse cancer cells. The results of these studies provide us with important information on the mechanistic role of 3-azidoWA on abrogation of invasion of cervical and prostate cancer cells that might be en-routed through extracellular Par-4.

## Materials and Methods

### Cell Culture and Antibodies

All cell lines were purchased from European Collection of Cell Culture (ECACC), Fetal Bovine Serum (FBS), RPMI-1640, Minimum Essential Medium (MEM), Penicillin G, Streptomycin, Trypsin-EDTA were obtained from Invitrogen Corp. 5-diphenyltetrazolium bromide (MTT), Paraformaldehyde, Crystal violet, Staurosporine, Phenylmethylsulfonyl fluoride (PMSF), Dithiothretol (DTT), NP-40, Protease Inhibitor Cocktail, Gelatin, Brij-35, Comassie Brilliant Blue (R-250), Brefeldin A (BFA), Tunicamycin, TRAIL, Annexin V-FITC apoptosis detection assay kit, Dimethylsulfoxide (DMSO), Bradford reagent was obtained from Sigma Chemicals Co. (St. Louis, MO) Propedium Iodide and Ultracruz DAPI mounting medium was obtained from Santa Cruz Biotechnology Inc.(Santa Cruz, CA), Z-VAD Pan caspase inhibitor, recombinant human VEGF-165 and recombinant human FGF (basic 146 aa) were obtained from R&D Systems (Minneapolis, MN). Cells were cultured in RPMI 1640/MEM containing 10% fetal bovine serum in presence of 70 mg/L penicillin and 0.1 g/L streptomycin and were incubated at 37°C with 95% air and 5.0% carbon dioxide. All cells were used in experiments during the linear phase of growth. Antibodies were obtained from following commercial sources: anti-Par-4, anti-MMP-2, anti-Akt, anti-ERK, and anti-TIMP-1 from Santa Cruz Biotechnology (Santa Cruz, CA), anti-p-Akt from and anti-caspase-3 (Cell Signaling) and anti-β-actin, anti-p-MAPK (Sigma Chemical, St. Louis, MO).

### Synthesis of 3-azido WA

Triethylamine was added to a solution of TMSN_3_ (1.2 equiv.) in dry methanol (3.0 ml) at room temperature (RT) to maintain the pH of 8.5. Withaferin A (1.0 equiv., 470 mg, and 1.0 mmol) was separately dissolved in dry methanol (2.0 ml) and the solution was added to the methanolic solution of TMSN_3_ and kept for the 3.5 h. The progress of the reaction was monitored through TLC. After the completion, the reaction mixture was dried completely, dissolved in water (5.0 ml) and extracted with CHCl_3_ (10 ml) three times to obtain the product 3-azidoWA. For 3-azidoWA treatment, 3-azidoWA was dissolved in 100% DMSO and diluted with culture medium so that the working concentration of DMSO was less than 0.2%.

### Clonogenic Assay

The assay was performed according to the previously described method [10]. Briefly, HeLa cells were plated at a seeding density of (1×10^3^ cells/well) in 6 well tissue culture grade plates. After 24 h the culture medium was changed and new medium was added and cells were exposed to various concentrations of 3-azidoWA/vehicle DMSO for 5 days at 37°C incubator in 5% CO_2_. Later on, the obtained colonies were fixed with 4% paraformaldehyde and were stained with 0.5% crystal violet solution. The colonies from the plates were counted and averaged from the observed fields randomly (n = 3) and photographed with Olympus c-7070 wide 700 M inverted microscope camera.

### Cell Proliferation Assay

The cell viability was determined by standard MTT dye uptake method [10]. Briefly, HeLa, PC-3, A549 and DU-145 cells (3×10^3^ cells/well) were plated into a 96 well tissue culture plate and treated with different concentration of 3-azidoWA in triplicate so that the final concentration of DMSO solvent was 0.2%. After 48 h incubation, MTT solution was added and cells were cultured for another 4 h at 37°C in 5.0% CO_2_ incubator. The amount of colored formazan derivative was determined by measuring optical density (OD) using TECAN microplate reader (Infinite M200 PRO) at 570 nm. The percentage viability was determined according to the protocol described [10].

### Scratch Motility (Wound Healing) Assay

The assay was performed as described previously [4]. Briefly, HeLa and PC-3 cells were plated in a 6 well plate at a concentration of (5.5×10^5^ cells/well) and allowed to form a confluent monolayer for 24 h, it was then serum starved for 24 h. After that the monolayer was scratched with a sterile pipette tip (200 µL), washed with serum free medium to remove floated and detached cells and photographed (time 0 h). Cells were successively treated in medium containing low serum (1.0%) in presence of different concentrations of 3-azidoWA (0.25, 0.50, and 0.75 µM) along with vehicle DMSO for 24 h. Wounded areas were progressively photographed with Olympus c-7070 with 700M camera (100x magnification).The percentage of wound closure was estimated by the following equation: wound closure % = [1-(wound area at t_1_/wound area at t_0_) x 100% ], where t_1_ is the time after wounding and t_0_ is the time immediately after wounding.

### Immunoblotting

HeLa or PC-3 cells (1×10^6^ cells) were incubated overnight and exposed to different concentrations of 3-azidoWA along with DMSO as vehicle. Cells were accordingly rinsed with PBS, trypsinized and collected after 24 h and then lysed with lysis buffer (HEPES 1.0 mM/L, KCl 60 mM/L, NP-40 0.3%, EDTA 1.0 mM/L, DTT 1.0 mM/L, Sodium orthovandate 1.0 mM/L, PMSF 0.1 mM/L, cocktail protease inhibitor). The cell extractions were centrifuged at 12,000 rpm for 10 min at 4°C. Protein concentration was determined by the standard Bradford method. Equal amount (20 µg) of protein from each sample was subjected to SDS-PAGE and proteins were transferred to membrane (Millipore), blocked with 5.0% (w/v) non-fat milk in PBS containing 0.1% Tween-20 and probed with relevant antibodies for 3 h at room temperature or overnight at 4°C. Subsequently blots were washed and probed with species specific secondary antibodies coupled to horseradish peroxidise. Immunoreactive proteins were detected by enhanced chemiluminescence plus (Amersham).

### Apoptosis, Annexin V-FITC and PI Staining

Following treatments with 3-azidoWA/vehicle DMSO, adherent cells (HeLa and PC-3) were harvested by trypsin digestion and washed twice with chilled PBS. Cells were fixed with 4% paraformaldehyde for 10 min followed by incubation with DAPI containing mounting medium for 15 min/RT in the dark and apoptosis was detected by fluorescence microscopy (100x magnification).The Annexin V-FITC experiments were performed using the Annexin V-FITC Apoptosis Detection Kit according to the manufacturer’s manual. Briefly, cell pellets were re-suspended in 600 µl binding buffer (10 mM HEPES [N-2- hydroxyethylpiperazine-N-2–ethanesulfonic acid], 140 mM NaCl, and 2.5 mM CaCl2, pH 7.4), and stained with 6.0 µl Annexin V-FITC and 10 µl propidium iodide staining solution for 20 minutes at room temperature in the dark. The samples were successively analyzed by FACS (BD FACS Aria II) using BD Diva software.

### Gelatin Zymography

The gelatinase activity of MMP-2 was assessed by method standardized previously. Accordingly, HeLa and PC-3 cells in sub confluent culture (∼70–80% cell density of confluent culture) were refreshed with new medium and kept for incubation with increasing concentrations of 3-azidoWA for 48 h. The conditional media obtained from both treated and untreated samples were employed for protein estimation and equal amount of total proteins (20 µg) were mixed with sample buffer (2.0% SDS, 25% glycerol, 0.1% Bromophenol blue and 60 mM Tris-HCl, pH 6.8). Gelatin zymography of the samples were carried out using 7.5% SDS-polyacrylamide gels containing 0.1% gelatine at 100V for 3 h at 4°C. Gels were accordingly rinsed with washing buffer (2.0% Triton X-100 in dd water) at room temperature to remove SDS followed by incubation overnight at 37°C in TCNB buffer (50 mM Tris-HCl, pH 8.0, 10 mM CaCl_2_, 0.02% NaN_3_). Gels were stained with Comassie blue R-250 (Sigma) (0.125% Comassie blue R-250, 50% methanol, 10% acetic acid) for 1–1.5 h and destained with destaining solution (20% methanol, 10% acetic acid, 70% dd water). Gelatinase activity was detected by observing unstained bands on a blue background on Comassie stained gel.

### Matrigel Invasion Assay

The effect of 3-azidoWA treatment on cell invasion was determined using BD Biocoat Tumor Invasion Assay System (BD Bioscience, Bedford, MA) according to the instruction of the manufacturer. Briefly, HeLa and PC-3 (1.25×10^6^) cells were cultured in the presence of 0.25, 0.50, and 0.75 µM 3-azidoWA or vehicle DMSO for 24 h in serum free media into the upper chambers/inserts, and the bottom wells were filled with chemo-attractant (complete media with 10% FBS). Cells were allowed to migrate at 37°C. After 24 h the Matrigel-coated polycarbonate filters were removed, the non-migrating cells were separated from the upper chamber with a cotton swab and the insert were fixed with methanol and stained with 0.1% crystal violet solution. For each replicate (n = 3), migration of the cells was quantified by counting the stained cells (cells per five fields) under inverted microscope.

### Transient Transfection

PC-3 and HeLa cells were cultured in appropriate medium as described in method section, transfected with GFP and GFP-Par-4 (generous gifts from Dr. Vivek Rangnekar, University of Kentucky, KY) using lipofectamine-2000, according to manufacturer’s instruction. Forty eight hours post-transfection, green-fluorescence cells were visualized under fluorescence microscope with camera and conditioned medium was employed for gelatin zymography.

### Immunocytochemistry

For immunostaining HeLa and PC-3 cells were plated on coverslips in 6-well plates at a seeding density of 0.5 x 10^6^ cells per well. After 24 h, cells were treated with 3-azidoWA (1.0 µM) or vehicle DMSO and positive control staurosporine (25 nM) in presence or absence of Pan-Caspase inhibitor for 48 h. After the incubation, cells were washed with PBS and fixed in 2.5% paraformaldehyde for 15 min at room temperature, permeabilized with 0.1% Triton X-100 in PBS for 5.0 min and was successively blocked in 0.5% BSA for 1 h. For detection of Caspase activity, the cells were incubated with Caspase-3 primary antibody (1∶5000 dilution in blocking buffer) for 1 h and accordingly washed three times with PBS, it was then incubated with Texas red (Invitrogen) conjugated secondary antibody (1∶10000 dilution) for 1 h and washed, mounted with ultracruz mounting medium and analyzed with Zeiss LSM-510 metaconfocal microscope. The images were captured at 63x magnification.

### In vivo Matrigel Angiogenisis Assay

Four to 6 week old C57/BL6J mice (Indian Institute of Integrative Medicine Central Animal House, Jammu, India) were maintained at 20–22°C on a 12 h light-dark cycle. Animal studies were performed in accordance with experimental protocols that were approved by the animal ethics committee of Indian Institute of Integrative Medicine, Jammu, India. Animals were injected subcutaneously, into the right flanks with 0.5 ml ice-cold Matrigel (BD Bioscience) supplemented with VEGF-A (250 ng/mL) and bFGF (500 ng/mL). Control mice were injected with Matrigel without VEGF-A and bFGF. At the end of each study animals were sacrificed to remove Matrigel plugs and photographs showing the extent of vascularisation was taken by using Nikon camera. The neovascularisation of Matrigel plugs was quantified by using 4 ml Drabkin’s reagent by adding well homogenated 20 µl of neovascularised Matrigel. After thorough mixing, absorbance was measured by spectrophotometer at wave length 540 nm to estimate hemoglobin. The hemoglobin estimation was calculated using formula Hb (g/dl) = absorbance of sample/absorbance of standard × concentration of standard.

A preliminary study was carried out to determine the time for optimal neovascularisation of Matrigel plugs to develop. In order to do this, plugs were removed from control mice (no added VEGF-A and bFGF) at the end of day 4, 7 and 11. The plugs that contained VEGF-A and bFGF were removed from mice at the end of days 2, 4, 7 and 11 after injecting Matrigel. Standardizing the optimal time for neovascularisation, a dose response study using 3-azidoWA was performed in which the compound was administered at 1, 10, 20, 30, and 50 mg/kg/d, i.p. for 3 days (8^th^, 9^th^, and 10^th^). At the end of duration, Matrigel plugs were removed for visualization and quantification. After identifying doses of 3-azido WA that inhibits vascularization, the number of doses to inhibit neovascularisation (preventive study) or disrupt established vasculature (treatment study) was investigated. For the preventive study mice were dosed with (30 mg/kg/d, i.p.) for 1–4 days after 24 h Matrigel injection. Seven days after injection of Matrigel plugs mice were sacrificed and the plugs removed for visualization and quantification.

For the treatment study, neovascularisation was allowed to develop over a 7-day period. Groups of animals were dosed with compounds on day 8 (1-day dosing) or days 8–10 (3 days dosing) at 30 mg/kg/d, i.p. The effects on established vasculature were assessed 11 days after the injection of the Matrigel plugs.

### Statistical Analyses

Data were expressed as mean ± SEM. Comparisons used Student’s *t* test. P<0.05 values were assigned significance.

## Results

### 3-azidoWA is an Anti-proliferative Agent and Induces Apoptosis in PC-3 and HeLa Cells

**Figure 1:**
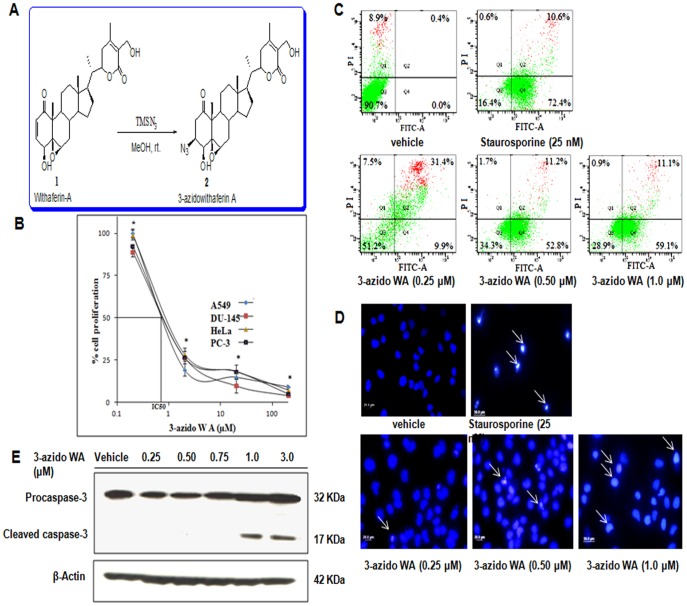
3-azidoWA is apoptosis inducing anti-proliferative agent. (A) Synthesis of 3-azidoWA. (B) Effects of 3-azidoWA on the cell proliferation of A549 (lung cancer), PC-3, DU-145 (prostrate cancer) and HeLa (cervical cancer) cell lines were determined by MTT assay. (C) 3-azidoWA along with vehicle DMSO and positive control staurosporine treated cells were analyzed by FACS, (as indicated); graphical representation of the results show proportion of non-apoptotic cells (Q3), early apoptotic (Q4), necrotic (Q1) and late apoptotic (Q2) cells. (D) HeLa cells (5×10^4^) were cultured in chamber slides and treated with 3-azidoWA (0.25, 0.50 and 1.0 µM) along with vehicle DMSO and positive control staurosporine (25 nM) for 24 h. After fixation, cells were stained with nuclear stain DAPI and photographed under fluorescence microscope (100x magnifications) to identify the apoptotic nuclei (white arrowheads). (E) HeLa cells were treated with increasing concentrations of 3-azidoWA as indicated, procaspase-3 and cleaved caspase-3 expressions were determined by Western blotting along with loading control β-actin.

Withaferin A is a potent cytotoxic agent and showed growth-inhibitory properties in tumor cell culture experiments [19], [26]. As good as parent molecule Withaferin A, the derivative of Withaferin, 3-azidoWA (Fig. 1, A) significantly exhibited anti-proliferative effect in a panel of cell lines tested (having IC_50_ within a range of 800 nM -1.0 µM) [25] (Fig. 1, B). For further confirmation of apoptosis by 3-azidoWA, we performed Annexin V-FITC staining of the 3-azidoWA treated HeLa cells. As depicted in Fig. 1, C 59.1% early apoptotic cells compared to 72.5% with 25 nM staurosporine, appeared when HeLa cells were treated with 1.0 µM 3-azidoWA for 24 h. By DAPI staining we observed that 1.0 µM of 3-azidoWA, not 0.5 µM induced apoptosis in Hela and PC-3 cells within 24 h of incubation as compared to positive control staurosporine (Fig.1, D). A residual passaging related early apoptoic cells were observed in FACS data (at lower concentration of 0.25 µM of 3-azidoWA treatment), however, higher concentration (1.0 µM) of 3-azidoWA, lead the cells towards apoptosis with chromatin condensation around the nuclear periphery accompanied by nuclear size reduction (white arrowheads, Fig. 1, D). Active caspase-3, quantified by western blot analysis showed cleavage of caspase-3 at 1.0 µM of 3-azidoWA treatment but not at sub-toxic doses (0.5 µM) (Fig. 1, E). Collectively, these results demonstrate that 3-azidoWA is a prospective cytotoxic and apoptosis inducing natural product derivative.

### 3-azidoWA Inhibits Cell Motility, Invasion and Colony Formation

**Figure 2:**
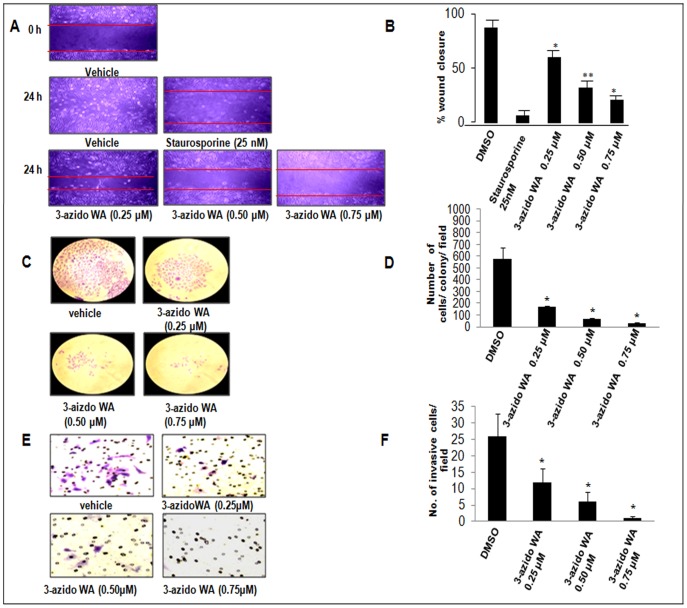
3-azidoWA inhibits motility, invasion and colony formation ability of HeLa cells. (A) HeLa cells (0.5×10^5^ cells/well) were grown to confluence in six well tissue culture plate and scratched with sterile tip; 3-azidoWA was added to cultures as indicated. Scratched areas were photographed (magnification 100x) at zero hour and then subsequently again 24 h later to assess the degree of wound healing. (B) The scratched areas were quantified in three random fields in each treatment, and data were calculated from three independent experiments. (C) HeLa (1×10^3^) cells/well were cultured and treated with various concentration of compound 3-azidoWA for 5 days at 37°C and then stained with crystal violet (for details see materials and methods), numbers of stained colonies were counted, photographed (100x) and (D) data were calculated from three independent experiments. Columns mean; bars SD of three independent experiments. P<0.05, **P<0.01 compared with untreated control. (E) Cell migration was determined via the modified Boyden chamber assay as described in Materials and Methods. HeLa cells (2×10^5^) were seeded in top chamber in the presence or absence of 0.25, 0.50, and 0.75 µM of 3-azidoWA. Cells were allowed to migrate for 24 h, at which point migratory cells on the bottom half of the insert membrane were stained with 0.1% crystal violet and counted under 200x magnification. (F) Invasive cells were counted using image software as the number of migrated cells per high-power field (HPF). Five fields were counted in triplicate (n = 3) from each insert. Cell images were obtained using microscope Nikon Eclipse E200 inbuilt with camera. Columns mean; bars SD of three independent experiments. P<0.05, **P<0.01 compared with untreated control.

As withanolides were known to inhibit invasion capability of cancer cells [27], we intended to study the effect of 3-azidoWA on the invasive potential of HeLa and PC-3 cells *in vitro.* Wound healing assays were used to determine whether sub-toxic concentration of 3-azidoWA could inhibit motility of Hela and PC-3 cells. After 48 h, cell monolayer’s were wounded, the vehicle DMSO treated cells had completely filled in the cleared area (Fig. 2, A), whereas treatment with 0.50 and 0.75 µM of 3-azidoWA significantly (p<0.05) inhibited motility of Hela and PC-3 cells, as did treatment with staurosporine (Fig. 2, A and B) (Fig. S1, A and B).

Colony formation ability of HeLa and PC-3 cells were attenuated by 3-azidoWA treatment in a dose dependent manner. As shown in Fig. 2, C sub-lethal doses of 3-azidoWA decreased colony formation ability of HeLa cells in statistically significant manner (P<0.05) (Fig. 2, D) (Fig. S1, C and D).

A critical event in tumor invasion and metastasis is the ability of tumor cells to invade through the extracellular matrix, allowing tumor cells to move beyond the confines of primary tumor environment [28]. To examine the effect of 3-azidoWA on cell invasion, Boyden chamber invasion assay was carried out to determine the ability of HeLa and PC-3 cells to invade through biological matrices *in vitro*. As shown in Fig. 2, E and F, (Fig. S1, E and F, P<0.01) treatment with 3-azidoWA (0.50 and 0.75 µM) inhibited cell invasion (P<0.05).To overrule the possibility of alteration of cell invasion due to programmed cell death, we carefully choose the physiologically relevant concentrations of 3-azidoWA. All these results collectively indicate that sub-toxic doses of 3-azidoWA alter the growth kinetics of HeLa and PC-3 cells. This could be a positive indicator for testing its antineoplastic activity in cervical and prostate cancer cells.

### Inhibition of Matrix Metalloproteinase 2 Gelatinase Activity and Expression by 3-azidoWA

**Figure 3:**
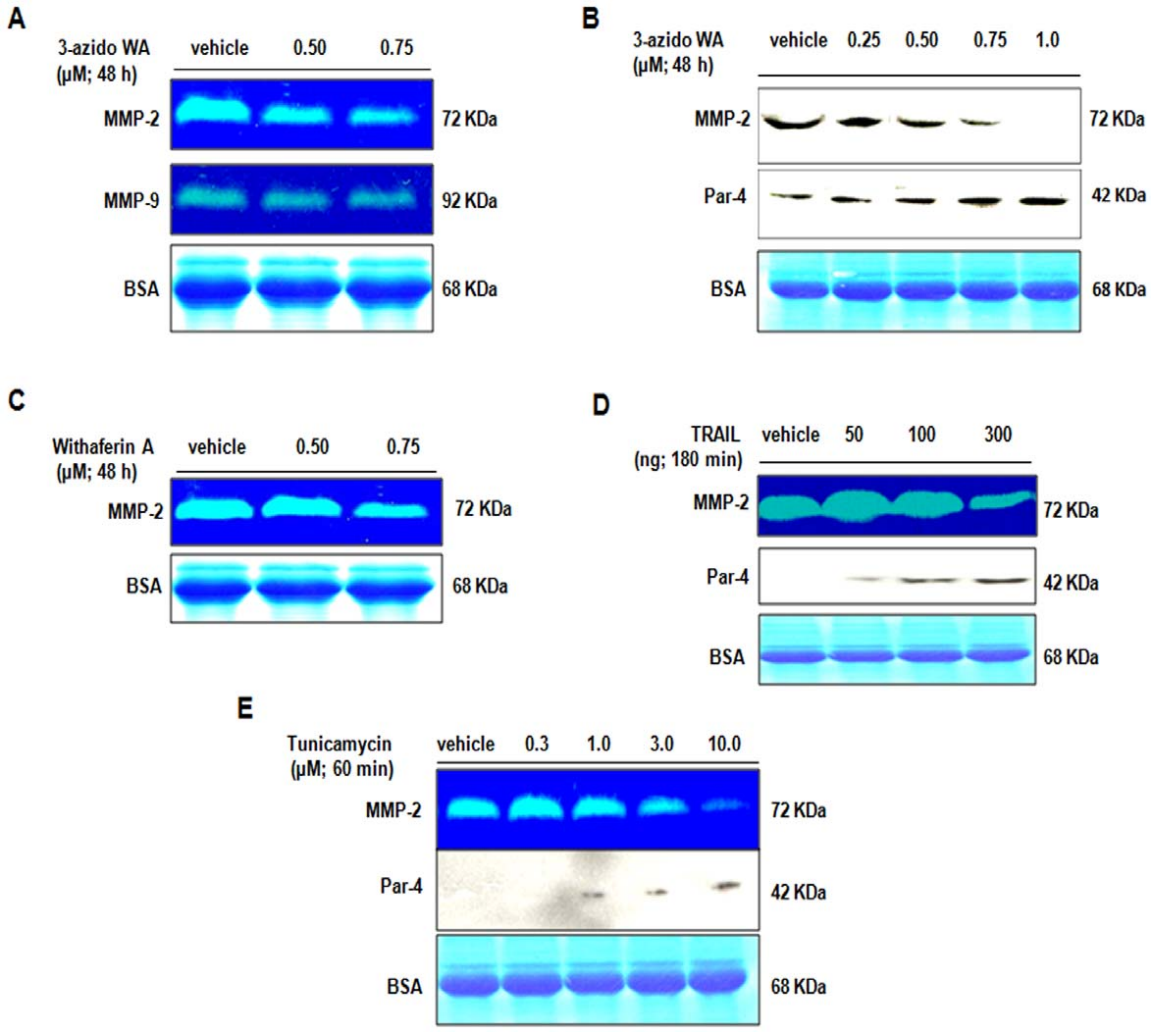
3-azidowithaferin induces extracellular Par-4 secretion and inhibits MMP-2. (A) HeLa cells were left untreated or treated with 0.50 µM and 0.75 µM of 3-azidoWA for 48 h, conditioned media was analyzed for MMP-2 & -9 gelatinase activity. (B) HeLa cells were left untreated or treated with 0.25, 0.50, 0.75 and 1.0 µM 3-azidoWA for 48 h, conditioned media obtained was employed for western blot analysis followed by Coomassie blue staining to reveal the 68 KDa BSA band for loading control. (C) HeLa cells were treated with various concentration of parent withaferin A for 48 h and the activity of MMP-2 was determined by gelatin zymography. (D) & (E) HeLa cells were treated with TRAIL for 180 minutes and with Tumicamycin for 60 minutes (as indicated). Conditioned media were subjected to gelatin zymography analysis for MMP-2 activity and Western blot analysis for extracellular Par-4, followed by coomassie blue staining for loading control.

Tumor cell invasion through matrix and tissue obstruction needs the combined effects of increased cell motility and controlled proteolytic degradation of matrix. High levels of MMP’s in tumor tissues have been correlated with cancer cell matrix degradation, invasion and metastasis [29]. As 3-azidoWA inhibited cell motility, we investigated whether 3-azidoWA exerts anti-gelatinase activity. As seen in Fig. 3, A and B (Fig. S2, A), upper row, increasing concentration of 3-azidoWA selectively abrogated gelatinase activity and expression of 72 KDa MMP-2 band as confirmed by gelatin zymography and Western blot analysis. The effect of MMP-2 inhibition by 3-azidoWA was more pronounced than parent molecule withaferin A (Fig. 3, C) (Fig. S2, B).Further we examined whether 3-azidoWA could inhibit other gelatinase (MMP-9) and results revealed that 3-azidoWA specifically block the MMP-2 activity in HeLa and PC-3 cells, but not MMP-9 (Fig. 3, A middle row) (Fig. S2, A middle row). Together, these findings suggest that 3-azidoWA strongly and selectively abrogates MMP-2 expression and activity in a dose dependent manner.

### 3-azidoWA Induces Extracellular Par-4 Secretion by Classical Pathway

**Figure 4:**
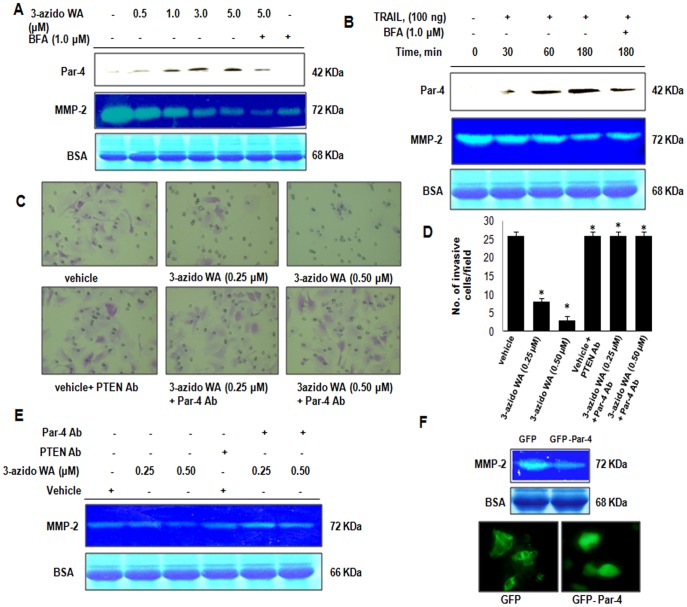
3-azidoWA induced extracellular Par-4 abrogates invasion **of PC-3 cells.** (A) PC-3 Cells were left untreated or pretreated with BFA (1.0 µM) for 120 min, then treated with 3-azidoWA as indicated for 48 h. Conditioned media were prepared, and subjected to gelatin zymography for MMP-2 activity and western blot (WB) analysis for Par-4, followed by Coomassie blue staining for loading control. (B) PC-3 cells were left untreated or pretreated with BFA (1.0 µM) as indicated, then exposed to TRAIL (as indicated). Conditioned media were prepared, and subjected to gelatin zymography for MMP-2 and western blot (WB) analysis for Par-4. (C) & (D) Cell migration was determined by the modified Boyden chamber assay as described in materials and methods. HeLa cells (2×10^5^ cells/well) were seeded in top chamber in the presence of 0.25 and 0.50 µM of 3-azidoWA along with untreated in upper and lower lane. Cells were allowed to migrate for 24 h, at which point migratory cells on the bottom half of the insert membrane were stained with 0.1% crystal violet and counted under 200x magnification and in lower lanes Par-4 antibody was added in 0.25 and 0.50 µM 3-azidoWA treatment group and control PTEN antibody was added in untreated group and further incubated for 24 h. Migratory cells were counted using image software as the number of migrated cells per high-power field (HPF). Five fields were counted in triplicate from each insert of three independent experiments (SD. *, P<0.05). Migrated cell images were obtained using microscope Nikon Eclipse E200 inbuilt camera (200x magnification). (E) Zymography was performed from conditional media collected from cells treated with 3-azidoWA (0.25 and 0.50 µM) along with untreated and 3-azidoWA (0.25 and 0.50 µM) + Par-4 antibody, corresponding untreated + PTEN antibody**.** Coomassie stain of BSA confirms equal loading (lower band). (F) PC-3 cells were transiently transfected with GFP and GFP-Par4, 48 h post-transfection conditioned media were analyzed through gelatin zymography, followed by coomassie blue staining to reveal the 68 KDa BSA band for loading control and green fluorescence protein expressions were checked under fluorescence microscope and photographed (100x). Columns mean; bars SD of three independent experiments. *P<0.05, compared with untreated control.

As couples of apoptosis inducing agents facilitate the secretion of extracellular Par-4 [3], we sought to examine a panel of medicinal plants derived pure natural products that could promote apoptosis and/or enhance secretion of Par-4 (data not shown). Surprisingly we found that as low as 0.5 µM (much below cytotoxic dose) of 3-azidoWA, but not the parent molecule Withaferin A (data not shown) induced the extracellular Par-4 secretion in dose dependent manner (Fig. 3, B middle row), verified by Western blot. Moreover, we observed 3-azidoWA treatment augmented the secretion of extracellular Par-4 in the conditional media that essentially mimicked the effects of 300 ng TRAIL (TNF- related apoptosis inducing ligand) and 1.0 µM of Tunicamycin treatment (Fig. 3, D and E middle row). To assess whether this secretion of Par-4 occurred via the classical BFA-sensitive pathway involving the ER/golgi route, we terminated protein trafficking from the ER to golgi with Brefeldin A (BFA) and found secreted Par-4 level was decelerated in the conditioned media after 3-azidoWA and TRAIL treatment in presence of Brefeldin A (Fig. 4, A and B upper row). Here, we suggest that 3-azidoWA induces extracellular Par-4 secretion by classical BFA sensitive pathway.

### Depletion of Extracellular Par-4 Restores Cell Invasion

Due to its immense physiological functions reported [30], Par-4 contributes major role in tumor regression and inhibition of NFκB activity [31]. Hence, we rationally examined the anti-metastatic role of tumor suppressor secretory Par-4. We pre-treated conditioned medium obtained from 3-azidoWA treated (48 h) HeLa cells with control PTEN antibody and Par-4 neutralizing antibody for 30 min and then added into the upper well of Boyden chamber containing Hela cell suspension. Interestingly these results showed cells pre-treated with Par-4 neutralizing antibody invaded through membrane in presence of 3-azidoWA compared to control PTEN antibody pre-treated cells (Fig. 4, C). The number of migrated cells received Par-4 antibody pre-treatment was highly invasive and as good as vehicle + PTEN antibody treated control cells (Fig. 4, D).

### Inhibition of MMP-2 by 3-azidoWA is Mediated through Extracellular Par-4

To investigate the role of extracellular Par-4 which is known to promote apoptosis [3], we examined whether there is any inter-relation between two physiologically important secretory proteins Par-4 and MMP-2. Thus, we depleted the secretory Par-4 in the conditioned medium (3-azidoWA treated well) by using Par-4 neutralizing antibody. The neutralization of extracellular Par-4 restored MMP-2 level compared to the control antibody (PTEN) treatment (Fig. 4, E). Further we verified the ectopically expressed Par-4 could inhibit MMP-2, as over expressed Par-4 was secreted spontaneously in the conditioned media [3]. We demonstrate that secreted GFP Par-4 not GFP inhibited MMP-2 gelatinase activity (Fig. 4, F) in PC-3 cells. Collectively these results indicate that 3-azidoWA blocks MMP-2 expression in a dose dependent manner and that may mediate through extracellular Par-4.

### Inhibition of MMP-2 Activity by 3-azidoWA is Independent of Apoptosis

**Figure 5:**
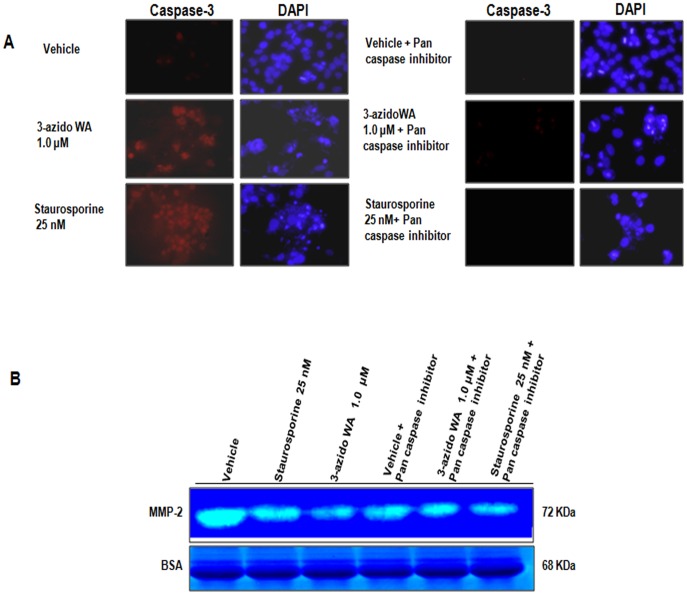
3-azidowithaferin induce MMP-2 inhibition is apoptosis independent. (A) HeLa cells (0.5×10^6^ cells/well) were plated in cover slip in 6 well tissue culture plates and were treated with 1.0 µM 3-azidoWA along with vehicle and staurosporine (25 nM) in presence (right panel) and absence (left panel) of Pan Caspase inhibitor. After 24 h treatment cells were washed, fixed and immunostained with caspase-3 antibody (Texas red), counterstained with DAPI (blue) to score apoptotic nuclei and analyzed random fields with Zeiss LSM-510 metaconfocal microscope and images were captured at 63x magnification. (B) Conditional media collected from above experimental group were employed for MMP-2 activity by zymography.

We further envisaged into the direct or indirect correlation of 3-azidoWA induced MMP-2 inhibition and concurrence of apoptosis. Previous studies have demonstrated the extrinsic pathway of apoptosis was induced by extracellular Par-4 [3] and siRNA mediated knock down of MMP-2 sensitized lung cancer cell apoptosis [32]. Therefore, we treated HeLa cells with vehicle, 1.0 µM of 3-azidoWA and 25 nM staurosporine in presence or absence of caspase inhibitor and conditioned medium was employed for analyzing MMP-2 activity. As shown in Fig. 5, A treatment of HeLa cells with 1.0 µM of 3-azidoWA and positive control staurosporine (25 nM) induced apoptosis, which was quantified by active caspase-3 staining and when apoptosis was blocked by Pan caspase inhibitor, MMP-2 activity level remained same as 1.0 µM 3-azidoWA alone treatment lane (Fig. 5, B). Our results suggest that inhibition of gelatinase activity of MMP-2 by 3-azidoWA is independent of apoptosis.

### 3-azidoWA Abrogates Akt-ERK Phosphorylation and Upregulate TIMP-1 Expression

**Figure 6:**
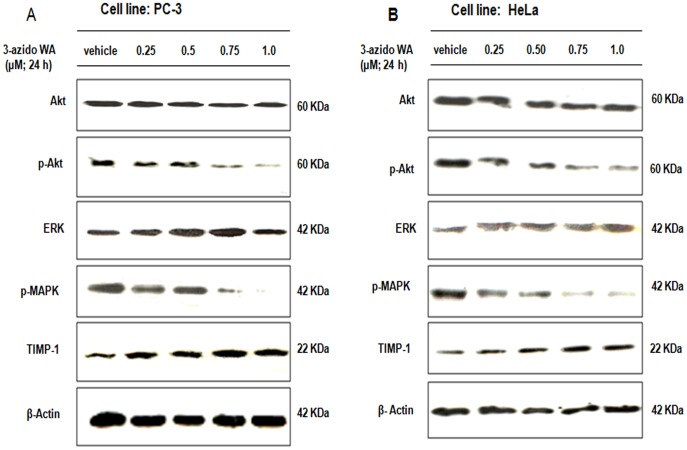
Effect of 3-azidoWA on phosphorylation of Akt, ERK and expression of TIMP-1. (A) HeLa and (B) PC-3 cells were treated with increasing concentrations of 3-azidoWA as indicated, phosphorylated and total Akt, ERK expressions were determined by Western blotting. TIMP-1 expression was verified by same method.

The importance of PI3K/Akt, ERK signaling pathways have been extensively studied in cancer cells invasion [33], [34], we seek to determine the effect of 3-azidoWA on these signaling pathways. Our results revealed that Akt and ERK phosphorylations were reduced when HeLa and PC-3 cells were treated with increasing concentration of 3-azidoWA respectively (Fig. 6, A and B). On the other hand 3-azidoWA treatment elevated the expression of TIMP-1 which blocked the proteolytic potential of MMP-2 expression in both cell lines, in dose dependent manner (Fig 6, A and B). Collectively these results demonstrate that 3-azidoWA inhibited the Akt and ERK phosphorylation in cervical and prostate cancer cells and augmented TIMP-1 expression.

### 3-azidoWA Inhibits *in vivo* Angiogenesis

Tumor angiogenesis provides oxygen, nutrients, and main routes for tumor growth, invasiveness, and metastasis and acts as a rate-limiting step in tumor progression [35]. Initial study was carried out to determine the time required for optimum neovascularisation of Matrigel lacking VEGF and bFGF. As shown in (Fig. S3, A) for animals injected with Matrigel with out VEGF and bFGF, minimal vascularisation was noticed and there was no increase in hemoglobin level of Matrigel plugs when retrieved from animals over a 7-11-days period. On the other hand plugs containing VEGF and bFGF revealed a marked degree of neovascularisation (n = 5, P<0.05) with maximum increase being achieved by 7-day period.

**Figure 7:**
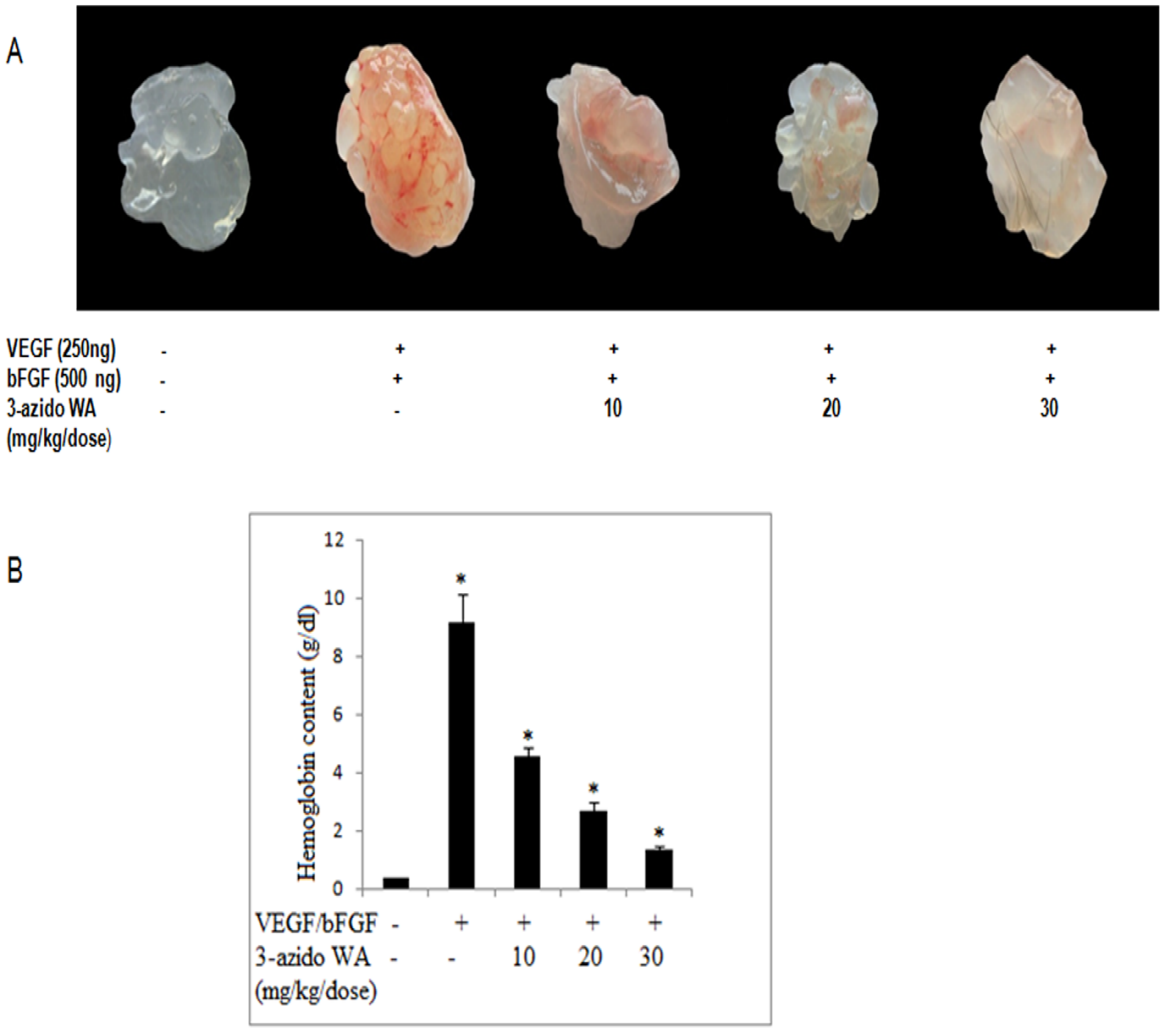
The macroscopic appearance of angiogenesis in Matrigel plugs. (A). Preventive study: The effect of dosing with 3-azido WA (30 mg/kg/d, i,p.) for 1–4 days on neovascularisation of Matrigel plugs. After dosing for 1–4 days plugs were removed on day 7 after Matrigel injection for visualisation and quantification of angiogenesis. Representive photographs of plugs from groups of five animals are shown. Quantification of angiogenesis within Matrigel plugs show (n = 5, P<0.05) compared with VEGF +bFGF. (B). Treatment study: The effect of dosing with 3-azido WA (30 mg/kg/d, i,p.) on established Matrigel plug vasculature. Neovascularisation was allowed to develop for 7 days animals were dosed for 1 day or daily for 3 days. Plugs were removed at the end of day 11 for photography and quantification after plugs were removed. Quantification of angiogenesis within Matrigel plugs shows (n = 5, P<0.05) compared with VEGF +bFGF.

To determine the effects of 3-azidoWA on VEGF/bFGF-induced angiogenesis *in vivo*, mice were dosed intraperitonally with 3-azido WA for 7 days, starting 24 h after Matrigel injection. Visual inspection of the plugs after removal revealed the marked increase in vascularization, seen as a deep red appearance that occurred in plugs of untreated animals (Fig. S3, B). Animals dosed with 3-azido WA 1,10, 20, 30 mg/kg/d, i,p dose but not with 50 mg/kg/d i,p (due to toxic effects and animals died) there was a marked reduction (n = 5, P<0.05) in the level of neovascularisation at 30 mg/kg/d as compared to untreated plugs (VEGF +bFGF). Having established that 30 mg/kg/d, i,p dose effectively abolished Matrigel plug vascularisation, this dose was used to investigate the number of doses required to prevent neovascularization (preventive study) or to disruptive established blood vessels (treatment study), as would be encountered in a therapeutic setting. For preventive study administration of 30 mg/kg/d of 3-azido WA daily for 1, 2, 3, or 4 days, 24 h after Matrigel injection, revealed that dosing for 4 days was sufficient to completely blocked plug neovascularisation as shown in Fig.7, A (n = 5, P<0.05). After letting neovascularisation become established over a 7-days period for treatment study, dosing for 3 days (i,e days 8–10) resulted in a marked significant reduction (n = 5, P<0.05) in plug vascularisation compared to control and one day (i,e day 8) dosing (Fig 7, B).Thus, these experiments clearly exhibit that 3-azidoWA may capable of suppressing VEGF induced neovessel formation and abolishe *in vivo* angiogenesis.

**Figure 8:**
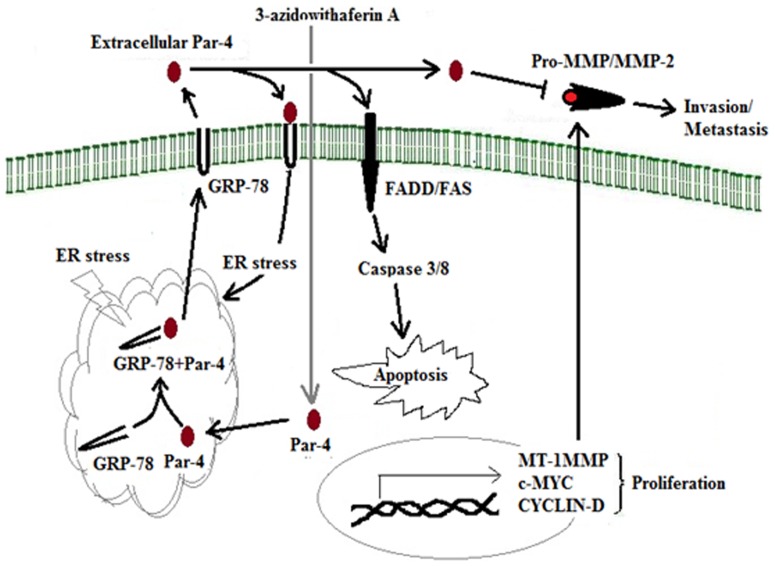
Schematic represents the extracellular Par-4 induction due to 3-azidoWA could abrogate invasion.

## Discussion

α-β-unsaturated carbonyl moiety exists in a plethora of natural products exhibiting cancer chemo-preventive and chemo-protective activities. Their biological activities are mostly attributed to their ability to act Michael acceptors. Currently, irreversible binding at active sites has been considered to be one of the reasons to drug resistance in cancer treatment [36]. Thus, in order to include α-β-unsaturated carbonyl group in drug development strategies, the potentially adverse effects must be circumvented. This can be achieved by fine tuning the reactivity of the α-β-unsaturated carbonyl unit leading to a high degree of specificity. Different approaches for comparing similar natural products and synthetic molecules have been followed in order to predict the reactivity of α-β-unsaturated carbonyl compounds. The α-β-unsaturated carbonyl moiety permits the direct adjustment of electrophilicity and reduction potential through modification of the peripheral substituents [36].

A structural modification of natural products represents an elegant approach towards finding new biological activities. In the light of promising therapeutic prospective of Withaferin A as an anticancer molecule reported by our institute [37], [38], we were keen to investigate modifications of the ring A with α-β-unsaturated carbonyl functionality. We envisaged that substitution of the α-β-unsaturated carbonyl system of Witheferin A at the β-position may be a practical approach to find new activities of the modified compounds as this will obviate irreversible covalent binding in active sites by biological nucleophiles, a reason behind drug resistance in cancer treatment [25]. This led to the discovery of pure compound 3-azidoWA (for compound purity supporting NMR, CMR, COSY data are provided in the supplementary section), a potent anticancer molecule than the parent withaferin A.

In the present study, we have identified a highly selective MMP-2 inhibitor, 3-azidoWA, a natural product withanolide derivative from traditional medicinal plant *W. Somnifera*. Though, withanolides have been well documented for diverse biological functions, *viz,* antiproliferative, anti-invasive, radiosensitizing and antiarthritic, here, for the first time, we uncover the target specific MMP-2 inhibition by 3-azidoWA in moderately aggressive cervical and prostate cancer cells. We demonstrate here that 3-azidoWA is a potent inducer of extracellular Par-4. Although, the intracellular Par-4 induction by Withaferin A had been empowered the apoptotic induction of these class compounds in several cancers [39], interestingly, we have explored the role of extracellular systemic Par-4 which was robustly increased in the conditioned media due to 3-azidoWA treatment as compared to Withaferin A treatment. Our data reveal that inhibition of MMP-2 expression and gelatinase activity by 3-azidoWA was extracellular Par-4 dependent.

Matrix metalloproteinases are the lead candidates to facilitate tumor invasion process by triggering a chain of signaling cascades and play crucial role not only in invasion, angiogenesis and metastasis, but also determine cancer cell transformation, growth, apoptosis and signal transduction [28], [40]. In the last twenty years majority of the MMP inhibitors showed lack of success in clinical trials because none of these synthetic/semi synthetic drugs were selective for specific MMP’s. Thus, learning lessons from looking back at previous failed attempts necessitate developing selective MMP inhibitors that should not have cross-reaction with other MMP’s [36]. Here we demonstrate that 3-azidoWA only selectively blocked MMP-2 activity and thus motility and invasion of diverse cell line in extracellular Par-4 dependent manner. By degrading the basement membrane, MMP’s are considered to accelerate cell motility in a stationary tumor cell and promote metastasis [41]. Mounting evidences have suggested that MMP’s not only function in cancer progression and metastasis but may also contribute to steps of cancer development [42]–[44]. These diverse activities may account for the role of MMP-2, a pivotal matrix metalloprotease that control cancer cell motility and invasion [45]. In the present study we have illustrated that non-toxic physiologically relevant doses of 3-azidoWA significantly (P<0.01) inhibited the migration and invasion of HeLa and PC-3 (Supplementary Fig. 1) cells when treated in a dose dependent fashion in Boyden chamber invasion assay.

In addition to degrading ECM components, MMP’s were shown to confer apoptosis resistance by modulating Fas-FADD mediated death signaling [46] and cleavage of Fas by MMP-2 resulted in decreased sensitivity of HT-29 colon carcinoma cells to Fas mediated apoptosis [32]. Extracellular Par-4, on the other hand has been documented to exert extrinsic apoptotic effect by activating downstream caspase-3 [3]. Earlier studies have shown that downregulation of MMP-2 with adenovirus mediated delivery of MMP-2 (ad-MMP-2) siRNA reduced invasion, migration and angiogenesis in A549 cells *in vitro* and inhibited tumor growth and metastasis *in vivo* [47]. Further the authors also revealed that Ad-MMP-2 mediated tumor growth inhibition occurred through cleavage of caspases-8,-9 and -3, and activation of FAS-mediated signaling pathway [32]. Consequently, we try to demonstrate here 3-azidoWA as well as TRAIL mediated inhibition of MMP-2 in cervical (data not shown) and prostate cancer cell line through induction of extracellular Par-4 which has been shown to induce extrinsic apoptotic pathway by FADD-caspase-8-caspase-3 activation [3]. Interestingly, we have observed that ectopically over expressed GFP-Par-4 which secreted spontaneously in the conditioned medium [3], significantly inhibited MMP-2 within 48 h of post transfection. Moreover, 3-azidoWA treatment caused activation of caspase-3 and inhibition of MMP-2 expression compared to vehicle treated cells and blocking of caspase-3 activation by caspase inhibitor could not restore the MMP-2 expression level. Hence, that inhibition of MMP-2 by 3-azidoWA was apoptosis independent. Interestingly, this azido derivative (3-azidoWA) did not confer any significant change in the cell proliferation data what we obtained and as reported earlier with parent Withaferin A [25], but it enhanced the biological activity of the said derivative tremendously. To address the rational discrimination of 3-azidoWA in controlling cell motility versus apoptosis we carefully choose a range of drug concentrations and observed that 0.5 µM 3-azidoWA did not contribute to DNA fragmentation (data not shown) but altered cell motility. Therefore, there may be a logical explanation that at a low dose of (0.5 µM) 3-azidoWA inhibited MMP-2, but had no effect on cleaved caspase-3 (data not shown) activation, but at the dose level of 1.0 µM, not only MMP-2 activity was strongly downregulated but also caspase-3 mediated apoptotic machinery might be unregulated via extracellular Par-4. Thus it might be possible that low dose of 3-azidoWA induced basal secretion of extracellular Par-4 that might be utilized to downregulate MMP-2 and additional increase of 3-azidoWA/prolong drug treatment might add excess pool of secretory Par-4 in the conditioned media that accelerated apoptosis by extrinsic pathway. But, how the intracellular Par-4 level was correlated with extracellular Par-4 secretion due to 3-zidoWA treatment is under current investigation in our laboratory.

The increased occurrence of spontaneous tumors in Par-4 null mice lends credence to the identification of Par-4 as a tumor suppressor protein but secreted form of Par-4 in the culture medium of both normal and malignant prostate cells has been characterized as a new physiologically relevant therapeutic target in cancer [3]. TRAIL on the other hand is one of the most studied and clinically relevant inducers of apoptosis [48] and enhanced TRAIL sensitivity has been observed in Jurkat cells after Par-4 transfection [49]. Here we have noticed a dose dependent increase in extracellular Par-4 level when HeLa/PC-3 cells were treated with TRAIL and Tunicamycin (with fairly high concentration of 300 ng/ml TRAIL and 1.0 µM Tunicamycin), which also inhibited MMP-2 gelatinolytic activity due to extrinsic apoptotic induction by secretory Par-4. In view of finding the role of 3-azidoWA induced extracellular Par-4, that escalated gradually with increasing concentration of 3-azidoWA (below toxicity), we found the concomitant weaken of MMP-2 band in the zymogram (having equal loading control BSA), that resembled anti-invasive property of conditioned media containing secretory Par-4. As cancer cells are prone to ER stress, 3-azidoWA treatment might add more ER stress to escalate the level of secretory Par-4 and promote signaling pathways that control cellular motility (Fig. 8). When these extracellular Par-4 in the conditional media was depleted with Par-4 neutralizing antibody, HeLa cells invaded through the membrane of the Boyden chamber (Fig. 4, C) and MMP-2 level was restored. Additionally, for further verification we diminished extracellular Par-4 secretion with BFA and found that depletion of extracellular Par-4 due to Brefeldin A treatment restored MMP-2 level compared to untreated vehicle. The inhibition of MMP-2 secretion by Brefeldin A might overcome by some mechanism need to be elucidated in future.

Angiogenesis is a crucial step in tumor progression and invasion regulated by VEGF [35]. MMP-2 plays a pivotal role in this process. Interestingly, our *in vivo* results reveal 3-azidoWA is capable of suppressing VEGF-bFGF induced neovessel formation *in vivo*. Note, the treatment with increasing concentration of 3-azidoWA almost abolished the new blood vessel formation in the plugs containing VEGF and bFGF (Fig 7, A). Due to the importance of TIMP-1 in cancer cells invasion [28], and angiogenesis, we have demonstrated that 3-azidoWA treatment augmented the expression of TIMP-1 which is a modulator of MMP-2 expression in both cell lines, in dose dependent manner. Moreover, MAPK and PI3K/Akt pathways also contribute pivotal role in tumor development, maintenance and angiogenesis [50], [51]. Therefore, we investigated the effect of 3-azidoWA on the activities of MAPK and PI3K/Akt signaling pathways. The results of our studies suggest that treatment with 3-azidoWA inhibited internal ERK and Akt phosphorylation in both HeLa and PC-3 (Supplementary Fig. 2, D) cells.

In conclusion, we exhibit a novel pathway of 3-azidoWA induced MMP-2 inhibition, mediated through pro-apoptotic secretory protein Par-4, secreted by a classical BFA sensitive pathway. We have verified the possibility of apoptotic induction and abrogation of invasion could be controlled independently/collaboratively by 3-azidoWA. Interestingly we disclose that 3-azidoWA mediated MMP-2 inhibition is apoptosis sovereign. How extracellular Par-4 modulates MMP-2 activity will be interesting topic to be explored in future. Taken together this report unveils a therapeutic potential of 3-azidoWA, derivative of dietary compound withaferin A to inhibit motility ability of cervical and prostate cancer cells through matrix metalloproteinase-2 by induction of extracellular Par-4, which has broader aspects of controlling tumorigenesis.

## Supporting Information

Figure S1 **(A) PC-3 cells (0.5×10^5^ cells/well) were grown to confluence in 6 well plate were scratched with sterile tip (200 µL) to create a wound, 3-azidoWA was added to cultures as indicated.** Scratched areas were photographed (magnification 100x) at zero hour and then subsequently again at 24 h to assess the degree of wound healing. (B) The scratched areas were quantified in three random fields in each treatment, and the data were calculated from three independent experiments. (C) PC-3 (1×10^3^) cells/well were cultured and treated with various concentration of compound 3-azidoWA for 5 days at 37°C and then stained with crystal violet (for details see materials and methods), numbers of stained colonies were counted, photographed (100x) and (D) data were calculated from three independent experiments. (E) Cell migration was determined via the modified Boyden chamber assay as described in Materials and Methods. PC-3 cells (2×10^5^) were seeded in top chamber in the presence or absence of absence of sub-toxic doses of 3-azidoWA. Cells were allowed to migrate for 24 h, at which point migratory cells on the bottom half of the insert membrane were stained with 0.1% crystal violet and counted under 100x magnification. (F) Invasive cells were counted using image software as the number of invasive cells per high-power field (HPF). Five fields were counted in triplicate from each insert. Cell images were obtained using microscope Nikon Eclipse E200 inbuilt with camera. Columns, means; bars SD of three independent experiments. *P<0.05, **P<0.01 compared with untreated control.
(TIF)

Figure S2 **(A) PC-3 cells were left untreated or treated with 0.50 µM and 0.75 µM of 3-azidoWA for 48 h, conditional media was analyzed for MMP-2 and -9 gelatinase activity.** (B) PC-3 cells were left untreated or treated with 0.25, 0.50, 0.75 and 1.0 µM 3-azidoWA for 48 h, conditioned media obtained was employed for western blot analyses followed by coomassie blue staining to reveal the 68 KDa BSA band for loading control. (C) PC-3 cells were treated with various concentration of parent molecule Withaferin A for 48 h and the activity of MMP-2 was determined by gelatin zymography.
(TIF)

Figure S3 **(A). Time course for neovascularisation in Matrigel plugs.** C57BL/J6 mice were injected subcutaneously with 0.5 ml Matrigel with or without VEGF+ bFGF. At the end of study plug were removed on days 2–11 from mice for visualisation and quantification of angiogenesis. (B). Effect of 3-azido WA on Matrigel plug neovascularisation. 3-azido WA was administered intraperitonially at the doses indicated for seven days starting 24 h after Matrigel injection. On day 8 animals were sacrificed and retrieve plugs for visualisation and quantification of angiogenesis within the Matrigel plugs achieved by haemoglobin estimation shows (n = 5,P<0.05) compared with the level of vascularisation in Matrigel plugs supplemented with VEGF +bFGF in animals. Representive of photographs of plugs from groups of five animals are shown.
(TIF)

Figure S4 1H NMR and 13C of 3-azido,2,3-dihydrowithaferin A.
(TIF)

Figure S5 1H-1H COSY of 3-azido,2,3-dihydrowithaferin A.
(TIF)

Figure S6 HSQC of 3-azido,2,3-dihydrowithaferin A.
(TIF)

Figure S7 HMBC of 3-azido,2,3-dihydrowithaferin A.
(TIF)
