# Supplementary figures and images for: A Novel MMP-2 Inhibitor 3-azidowithaferin A (3-azidoWA) Abrogates Cancer Cell Invasion and Angiogenesis by Modulating Extracellular Par-4
Source: PLoS One. 2012 Sep 4;7(9):e44039. doi: 10.1371/journal.pone.0044039 (PMC3433490; doi:10.1371/journal.pone.0044039)

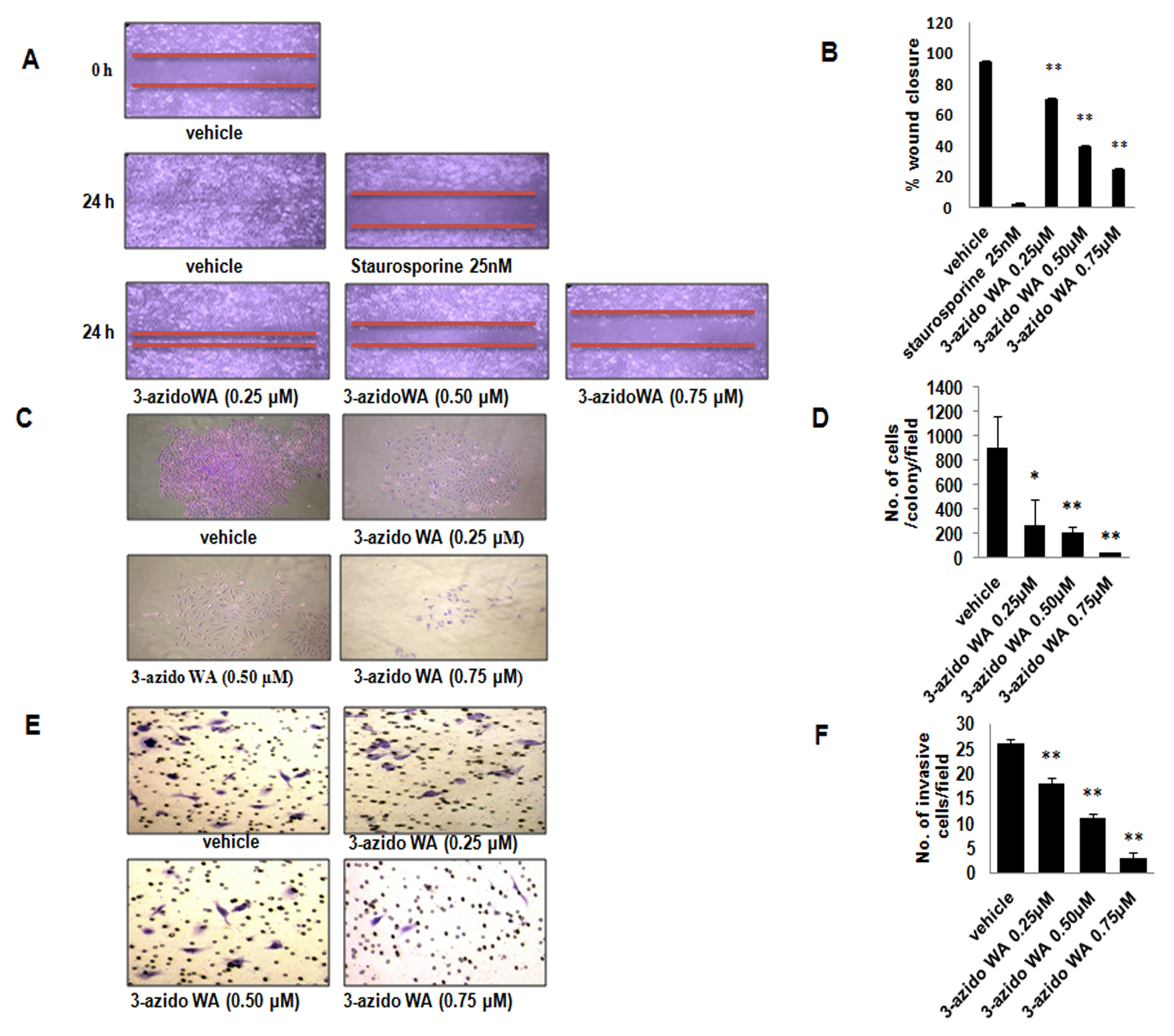

Supplement: Figure S1 — (A) PC-3 cells (0.5×10^5^ cells/well) were grown to confluence in 6 well plate were scratched with sterile tip (200 µL) to create a wound, 3-azidoWA was added to cultures as indicated. Scratched areas were photographed (magnification 100x) at zero hour and then subsequently again at 24 h to assess the degree of wound healing. (B) The scratched areas were quantified in three random fields in each treatment, and the data were calculated from three independent experiments. (C) PC-3 (1×10^3^) cells/well were cultured and treated with various concentration of compound 3-azidoWA for 5 days at 37°C and then stained with crystal violet (for details see materials and methods), numbers of stained colonies were counted, photographed (100x) and (D) data were calculated from three independent experiments. (E) Cell migration was determined via the modified Boyden chamber assay as described in Materials and Methods. PC-3 cells (2×10^5^) were seeded in top chamber in the presence or absence of absence of sub-toxic doses of 3-azidoWA. Cells were allowed to migrate for 24 h, at which point migratory cells on the bottom half of the insert membrane were stained with 0.1% crystal violet and counted under 100x magnification. (F) Invasive cells were counted using image software as the number of invasive cells per high-power field (HPF). Five fields were counted in triplicate from each insert. Cell images were obtained using microscope Nikon Eclipse E200 inbuilt with camera. Columns, means; bars SD of three independent experiments. *P<0.05, **P<0.01 compared with untreated control. (TIF) [file pone.0044039.s001.tif]

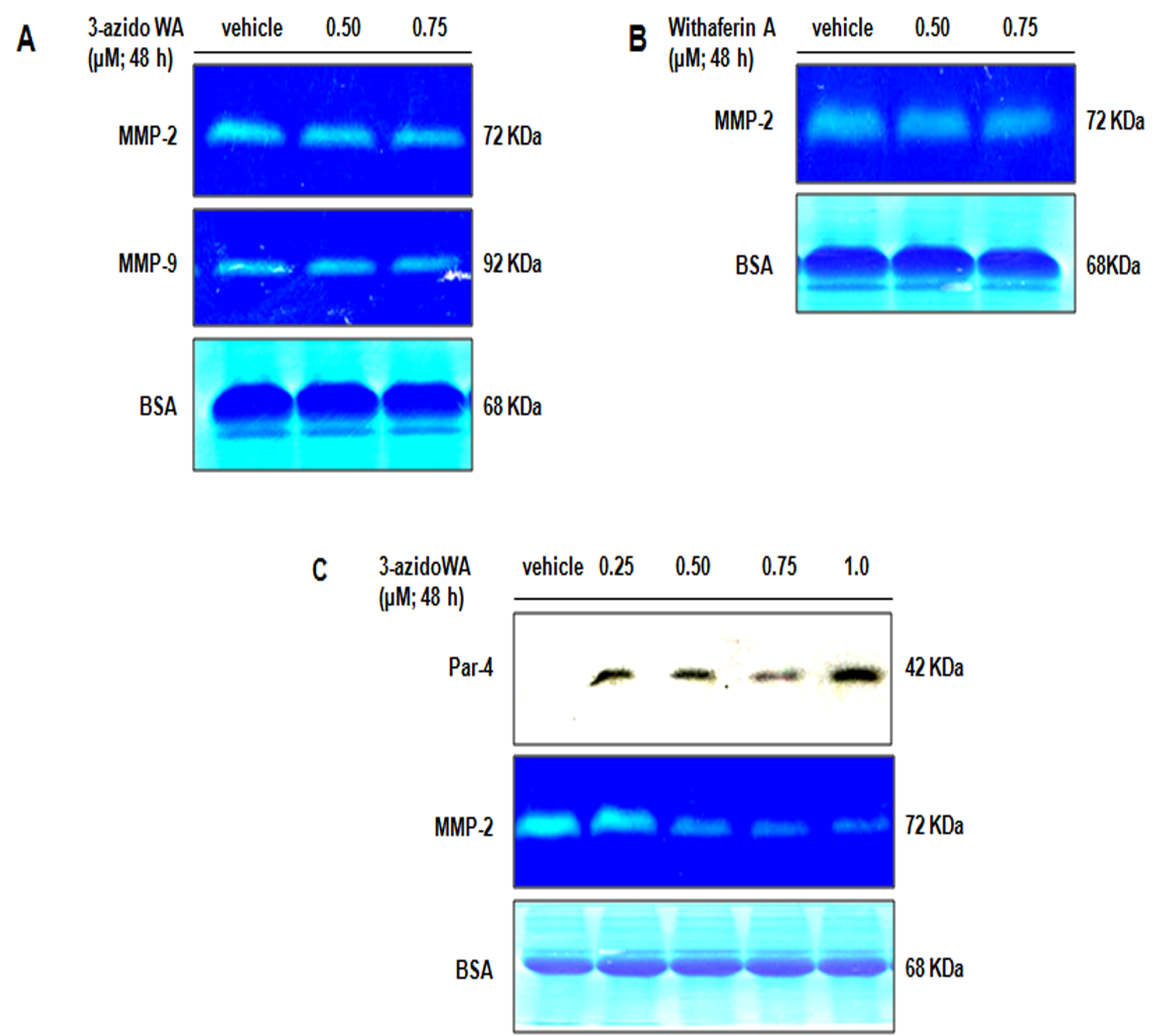

Supplement: Figure S2 — (A) PC-3 cells were left untreated or treated with 0.50 µM and 0.75 µM of 3-azidoWA for 48 h, conditional media was analyzed for MMP-2 and -9 gelatinase activity. (B) PC-3 cells were left untreated or treated with 0.25, 0.50, 0.75 and 1.0 µM 3-azidoWA for 48 h, conditioned media obtained was employed for western blot analyses followed by coomassie blue staining to reveal the 68 KDa BSA band for loading control. (C) PC-3 cells were treated with various concentration of parent molecule Withaferin A for 48 h and the activity of MMP-2 was determined by gelatin zymography. (TIF) [file pone.0044039.s002.tif]

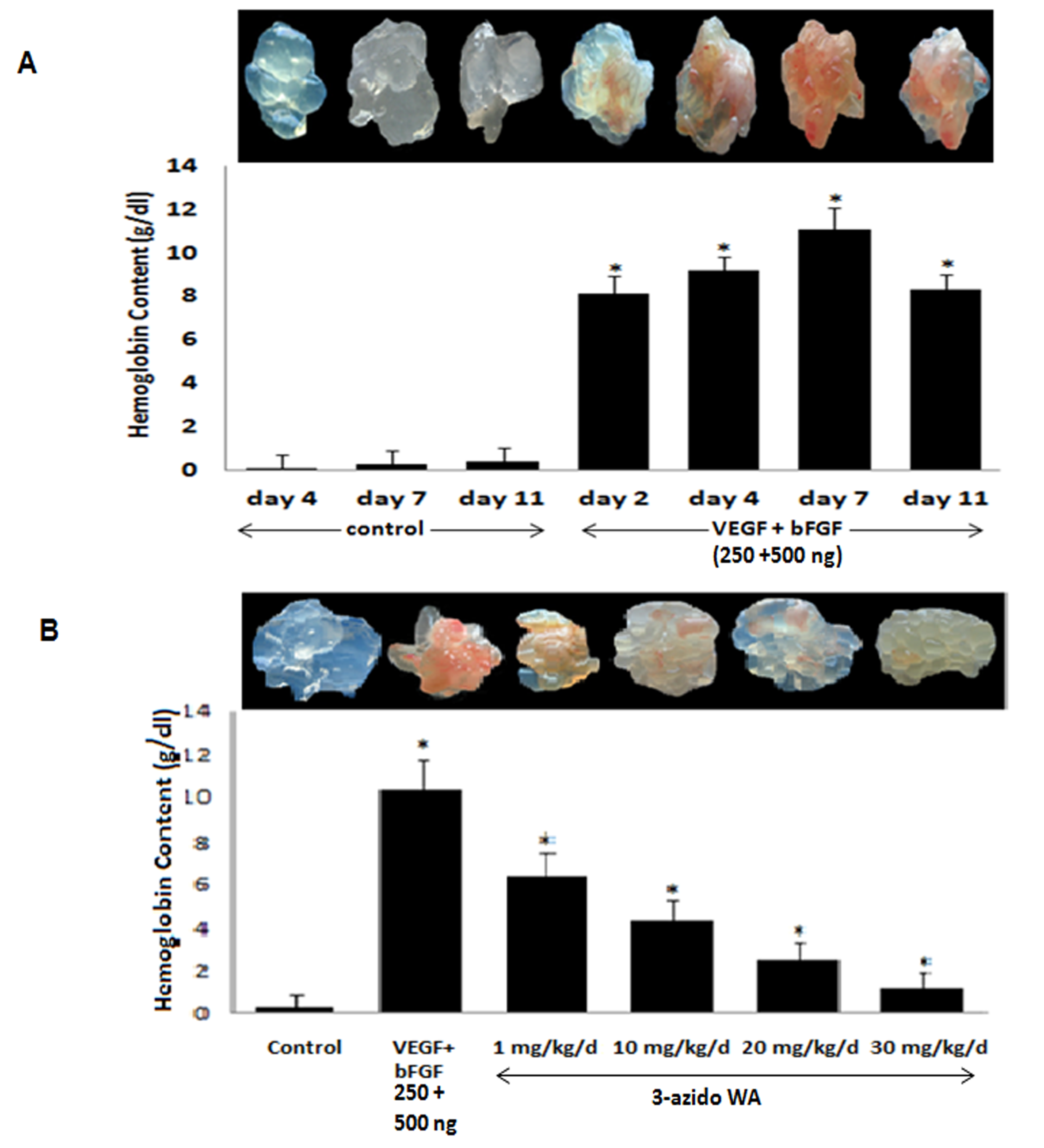

Supplement: Figure S3 — (A). Time course for neovascularisation in Matrigel plugs. C57BL/J6 mice were injected subcutaneously with 0.5 ml Matrigel with or without VEGF+ bFGF. At the end of study plug were removed on days 2–11 from mice for visualisation and quantification of angiogenesis. (B). Effect of 3-azido WA on Matrigel plug neovascularisation. 3-azido WA was administered intraperitonially at the doses indicated for seven days starting 24 h after Matrigel injection. On day 8 animals were sacrificed and retrieve plugs for visualisation and quantification of angiogenesis within the Matrigel plugs achieved by haemoglobin estimation shows (n = 5,P<0.05) compared with the level of vascularisation in Matrigel plugs supplemented with VEGF +bFGF in animals. Representive of photographs of plugs from groups of five animals are shown. (TIF) [file pone.0044039.s003.tif]

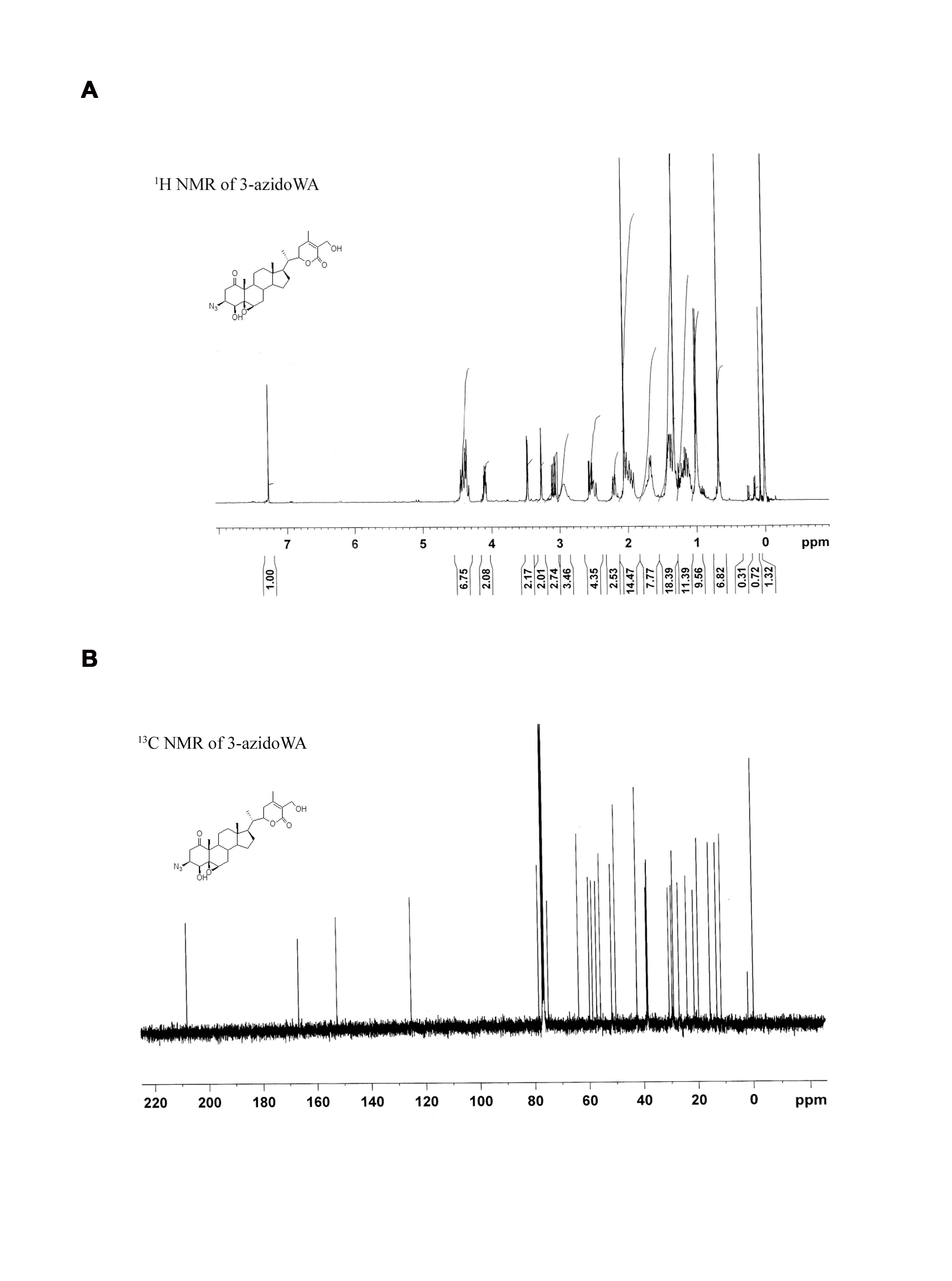

Supplement: Figure S4 — 1H NMR and 13C of 3-azido,2,3-dihydrowithaferin A. (TIF) [file pone.0044039.s004.tif]

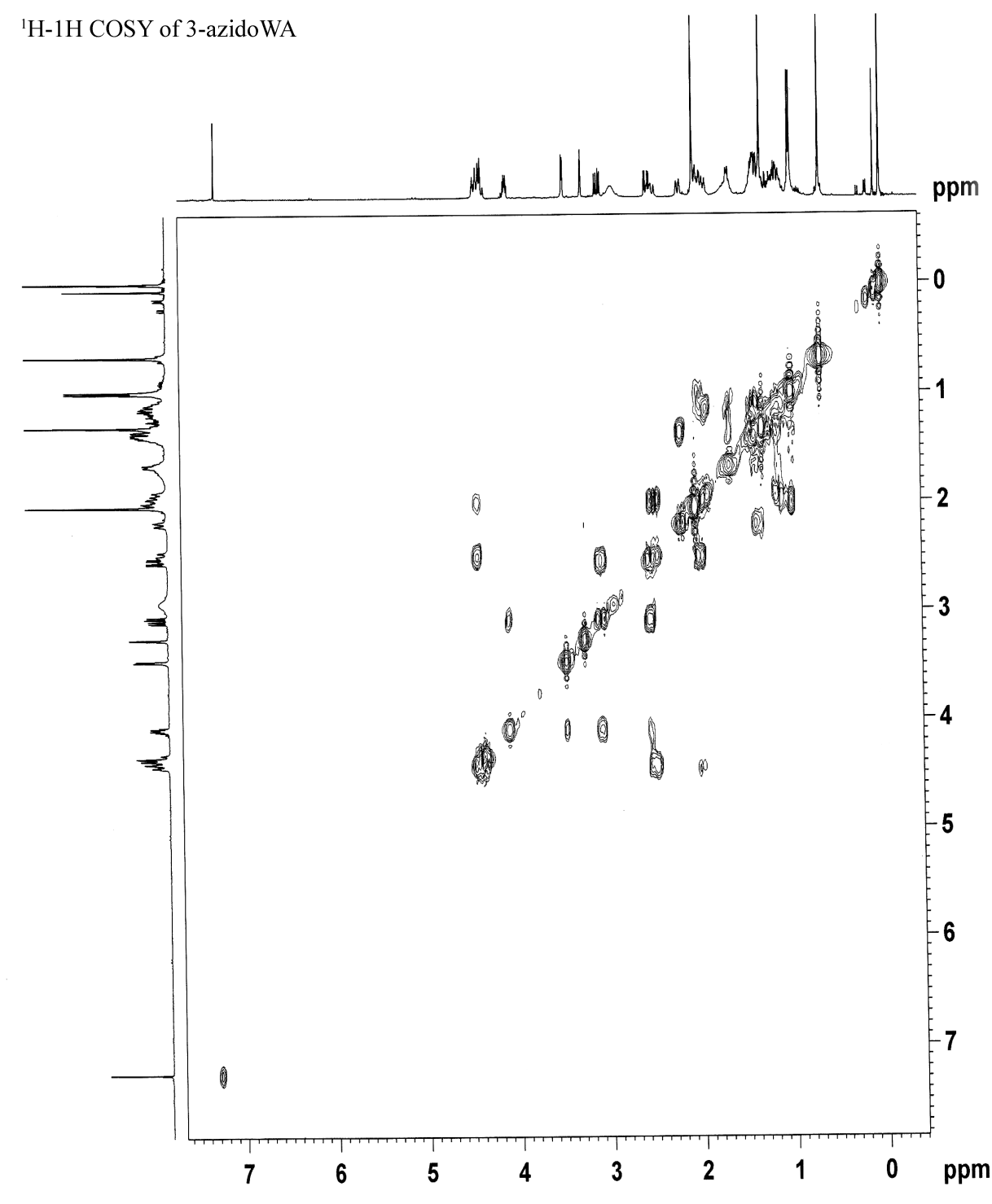

Supplement: Figure S5 — 1H-1H COSY of 3-azido,2,3-dihydrowithaferin A. (TIF) [file pone.0044039.s005.tif]

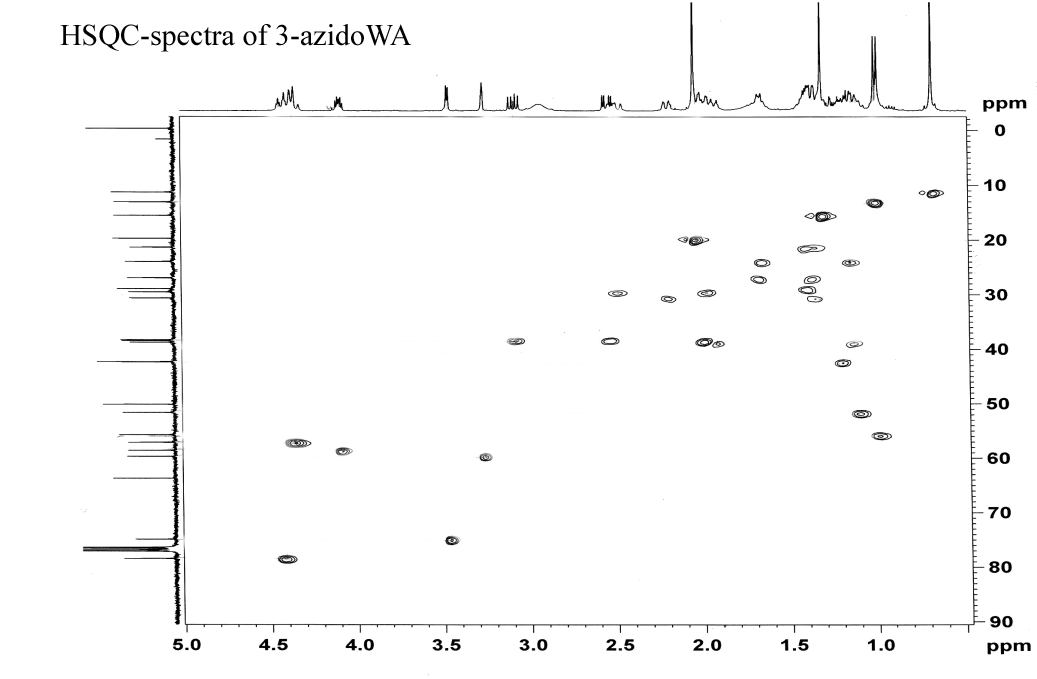

Supplement: Figure S6 — HSQC of 3-azido,2,3-dihydrowithaferin A. (TIF) [file pone.0044039.s006.tif]

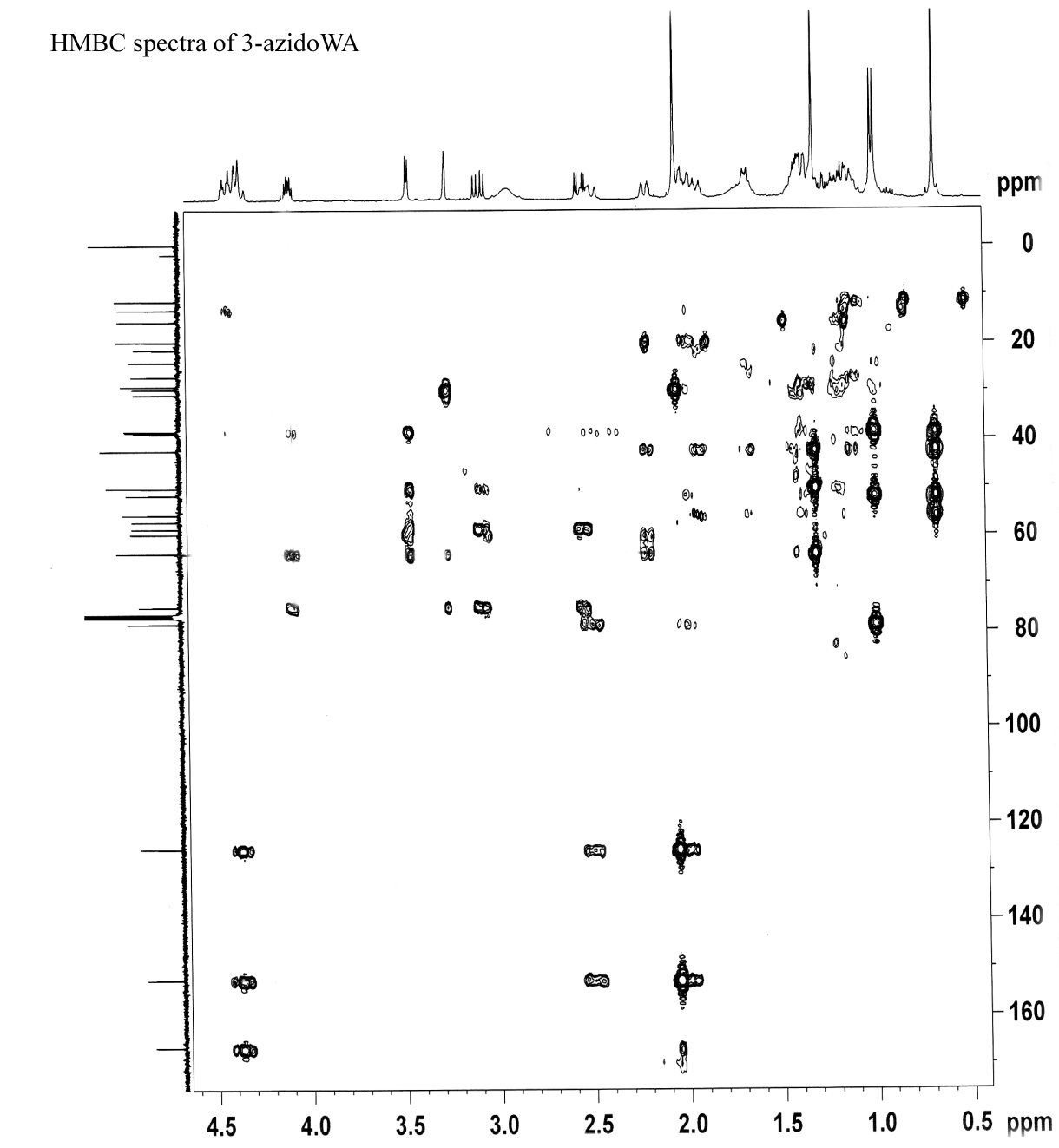

Supplement: Figure S7 — HMBC of 3-azido,2,3-dihydrowithaferin A. (TIF) [file pone.0044039.s007.tif]
